# Molecular mechanisms of polarized transport to the apical plasma membrane

**DOI:** 10.3389/fcell.2024.1477173

**Published:** 2024-09-26

**Authors:** Masataka Kunii, Akihiro Harada

**Affiliations:** Department of Cell Biology, Graduate School of Medicine, The University of Osaka, Osaka, Japan

**Keywords:** polarized transport, cell polarity, apical transport, unconventional transport, Rab8

## Abstract

Cell polarity is essential for cellular function. Directional transport within a cell is called polarized transport, and it plays an important role in cell polarity. In this review, we will introduce the molecular mechanisms of polarized transport, particularly apical transport, and its physiological importance.

## Introduction

**FIGURE 1:**
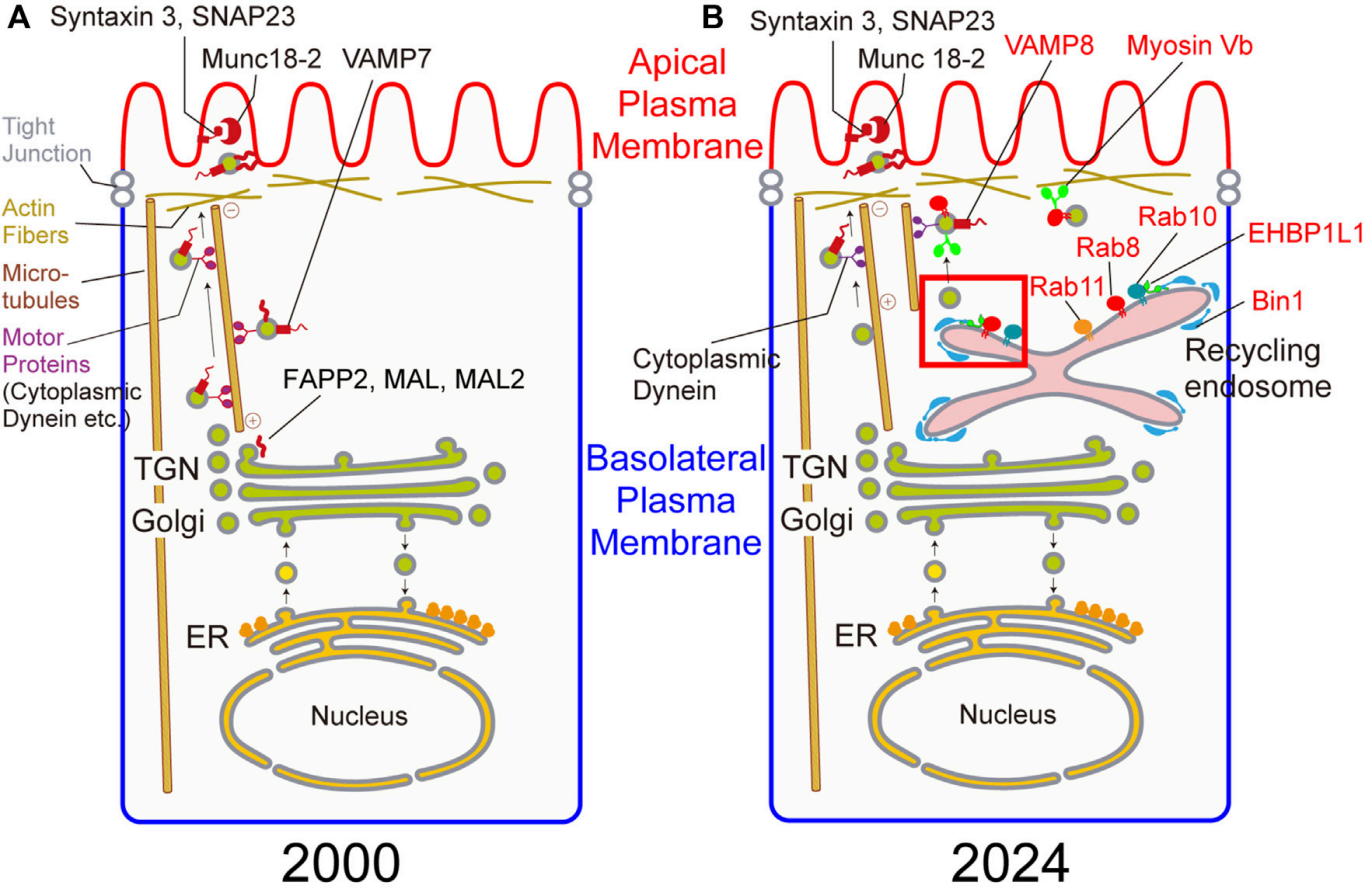
**(A)** Candidate genes involved in apical transport in 2000. **(B)** Genes involved in apical transport confirmed by gene knockout in 2024. Genes in red are newly identified genes in apical transport since 2000. Budding at the recycling endosome in a red square is magnified in Figure 2.

Cells have unique shapes. For example, epithelial cells that cover the lumen of epithelial tissues such as the intestine and the kidney have a directionality between the apical surface close to the lumen and the basolateral surface on the opposite side (Figure 1). These directions are called cell polarity. Cell polarity is not limited to epithelial cells, but exists in various cells. For example, neurons have two types of processes: that is, axons and dendrites. Thus, they can be considered to have cell polarity. In addition, lymphocytes are usually spherical and appear to have no directionality. However, when they attach to antigen-presenting cells and obtain information about antigens, polarity is required, i.e., an attached side and a non-attached side. Therefore, almost all types of cells have polarity.

In the intestine, cell polarity plays an important role in functions such as digestion and absorption of nutrients These functions as well as exocytosis of enzymes are performed at the apical plasma membrane. On the other hand, cell adhesion molecules (e.g., cadherins) and nutrient receptors (e.g., LDL receptor) are mainly localized at the basolateral plasma membrane. To generate and maintain cell polarity, selective transport to the apical or basolateral plasma membranes, collectively called polarized transport, is necessary.

In transport to the plasma membrane, transmembrane or secreted cargos are synthesized in the ER, transported to the Golgi. After glycosylation or other posttranslational modifications in the Golgi, cargos exit from the trans Golgi network or TGN. Some cargos directly go to the plasma membrane whereas other cargos go to the recycling endosome and then to the plasma membrane (Ang et al., 2004). In polarized epithelial cells, there are some specialized compartments for polarized transport, such as the apical recycling endosome (ARE), the apical sorting endosome (ASE), the basolateral sorting endosomes (BSE), the common recycling endosome (CRE) (Rodriguez-Boulan et al., 2005; Stoops and Caplan, 2014). In this review, we presented a simplified cartoon (Figure 1) to depict the organelles involved in the polarized transport because we have not determined the exact localization of the molecules in these specialized compartments.

During the vesicular transport including polarized transport, cargos synthesized in the ER have to be transported from the ER by vesicles budded from the ER exit sites. During this process, COPII (Sec13, 31, 23, 24) and their associated molecules (Sec16, Sar1, etc.) are known to be involved. After budding, vesicles are transported to the perinuclear Golgi by microtubule-dependent motor proteins, particularly cytoplasmic dynein. In the Golgi, COPI and associated molecules are involved in the intercisternal transport. Up to transport to the Golgi, molecular mechanisms are relatively clarified.

After modification such as glycosylation in the Golgi, cargos are sorted in the Golgi, the TGN, or the recycling endosomes. There are so many ambiguities during and after this sorting process. There are a number of candidate molecules involved after the sorting process as shown in the left figure of Figure 1 and Table 1 at the end of 20th century. Here, there are several types of molecules involved in polarized transport. Rab8, 10, and 11 belong to Rab family, one family of small GTPases. They are known to localize on the vesicles and the organelles to tether the donor membrane to the acceptor membrane. SNAP23, syntaxin3, VAMP7, and VAMP8 belong to SNARE family which are essential for fusing the donor membrane to the acceptor membrane. Munc18-2 are a regulatory protein for SNARE. During our research, other proteins have been identified to be involved in apical transport. EHBP1L1, Bin1, and dynamin1 are largely involved in the formation of apically-destined vesicles at the recycling endosomes. Also, a microtubule-dependent motor protein, cytoplasmic dynein, and an actin-dependent motor protein, myosin Vb, are important proteins for polarized transport to the apical plasma membrane. Other researchers pointed out the importance for lipids and glycosylation for polarized transport. For lipids, from many years ago, the raft, a lipid domain enriched with sphingomyelin and cholesterol are known to be involved in sorting apical cargos (Simons and Ikonen, 1997). In addition, family of phosphoinositides are selectively enriched in the apical or basolateral plasma membranes indicating their importance in polarized transport. Also, glycosylation has been known to be required for cargo sorting (Fiedler and Simons, 1995; Urquhart et al., 2005; Potter et al., 2006). Part of these molecules are shown in Figure 1. Current findings concerning these molecules are described in more detail in the following sections.

**TABLE 1:** The phenotypes of knockout mice of genes involved in apical transport. Yellow cells (lanes) shows the results from other laboratories.

Targeted gene	Type of knockout mouse	Phenotype	References
Rab8a KO	Systemic, intestine-specific (Villin-Cre)	Accumulation of apical proteins, microvillus trucation	Sato et al. (2007)
Rab8b KO	Systemic	No overt phenotype	Sato et al. (2014)
Rab8ab DKO + Rab10 KD	MEF (mouse embryonic fibroblast)	Reduction of number of cilia + MEFs	Sato et al. (2014)
Rab11a KO	Systemic	Embryonic lethal	Sobajima et al. (2014)
	Neuron-specific (Nestin-Cre)	No overt phenotype	Sobajima et al. (2014)
	Intestine-specific (Villin-Cre)	Accumulation of apical proteins, microvillus trucation	Sobajima et al. (2014)
Rab6a KO	Systemic	Embryonic lethal	Iwaki et al. (2020)
	Intestine-specific (Villin-Cre)	Accumulation of milk in lysosomes of enterocytes	Iwaki et al. (2020)
Rab6b KO	Systemic	No overt phenotype	Zhang et al. (2024)
Rab6ab DKO	Neuron-specific (Nestin-Cre)	Purterbation of neuronal polarity	Zhang et al. (2024)
EHBP1L1 KO	Systemic	Reduced enucleation efficiency, centronuclear myopathy	Wu et al. (2023)
Rab10 KD	Erythroblast	Reduced enucleation efficiency	Wu et al. (2023)
SNAP23 KO	Systemic	Embryonic lethal	Kunii et al. (2016)
	Exocrine pancreas-specific (Elastase-Cre)	Reduced exocytosis of secretory granules	Kunii et al. (2016)
	Endocrine pancreas-specific (Ins-Cre, Pdx1-Cre)	Increased exocytosis of insulin granules	Kunii et al. (2016)
	Neuron-specific (Nestin-Cre)	Purterbation of polarity of neuronal stem cells	Kunii et al. (2020)
VAMP7 KO	Systemic	Slight reduction in axonal length	Sato et al. (2011)
VAMP8 KO	Systemic	Reduced exocytosis in exocrine pancreas and kidney	Wang et al. (2004), Wang et al. (2010)
syntaxin3 KO	Systemic	Embryonic lethal	manuscript in preparation
	Neuron-specific (Nestin-Cre)	No overt phenotype	manuscript in preparation
	Intestine-specific (Villin-Cre)	Accumulation of apical proteins, microvillus trucation	manuscript in preparation
myosin Vb KO	Systemic, intestine-specific (Villin-CreERT2)	Accumulation of apical proteins, microvillus trucation	Cartón-García et al. (2015); Schneeberger et al. (2015)
MAL KO	Systemic	Paranodal malformation in the central nervous system	Schaeren-Wiemers et al. (2004)
MAL2 KO	Systemic	No overt phenotype	our unpublished observation
Annexin13b KO	Systemic	No overt phenotype	our unpublished observation
PKD1 KO	Systemic	No overt phenotype	Atik et al. (2014), Avriyanti et al. (2015)
PKD2 KO	Systemic	No overt phenotype	Atik et al. (2014), Avriyanti et al. (2015)
PKD1,2 DKO	Systemic	Embryonic lethal, neuronal polarity defect	Atik et al. (2014), Avriyanti et al. (2015)
FAPP1	Systemic	No overt phenotype	Venditti et al. (2019)
FAPP2	Systemic	No overt phenotype	D’Angelo et al. (2013)

*Yellow cells indicate results from other laboratories.

As shown in Figure 1, the biological significance of many genes previously known to be involved in polarized transport became obscure. The main reason for this comes from the experimental system used to verify the function of these molecules. Formerly, a number of researchers preferred to use MDCK cells, which are believed to be derived from distal tubules of canine kidney, to study the mechanism of cell polarity because cell polarity can be easily established *in vitro*. In the past, the plasma membrane of this cell line was solubilized, and antibodies or short oligonucleotides against molecules important for cell polarity were introduced into the cytoplasm to investigate the importance of these molecules (Huber et al., 1993). Previously, we generated knockout mice for the tau gene, which was thought to be necessary for axonal elongation based on results in cultured cells, but no major abnormalities were observed (Harada et al., 1994). From this experience, we felt it necessary to re-examine the results obtained in cell lines using knockout mice. In addition, as cell polarity is important for the development and functions of tissues, we generated and analyzed knockout mice for these molecules to investigate when and where they were required. As expected to some extent, the results obtained in cell lines could not be confirmed *in vivo* for many of the molecules (Figure 1; Table 1). However, we found that some molecules were indeed involved in cell polarity, and we were able to further investigate their molecular mechanism. Examples of these are the small GTP-binding proteins Rab8, 10, and 11, as well as the SNARE proteins syntaxin3 and SNAP23 which we already mentioned above. We will provide an overview of research in this field, especially on apical transport, focusing on the phenotypes of these knockout mice and the analysis of the molecules that bind to them (Figure 1).

## The apical and basolateral signals

Before explanation of each molecule for apical transport, it is necessary to briefly explain the sorting signals for apical and basolateral transport because it will give us reasons why apical transport has been difficult to analyze. Please refer to other good reviews for more information (Stoops and Caplan, 2014; Rodriguez-Boulan and Macara, 2014)

Previous studies have identified the basolateral signals (tyrosine-based, dileucine, and monoleucine signals) (Matter et al., 1992; Hunziker and Fumey, 1994; Wehrle-Haller and Imhof, 2001) at the cytoplasmic tails of the cargoes. An adaptor protein AP1B binds to many of these signals (Ohno et al., 1999; Sugimoto et al., 2002). Thus, clathrin-coated vesicles which includes AP1B, are considered to be involved in the transport of basolateral cargos (Deborde et al., 2008).

In contrast, for apical sorting signals, GPI-anchor, N- and O- glycosylation, and transmembrane domains (Lisanti and Rodriguez-Boulan, 1990; Fiedler and Simons, 1995; Urquhart et al., 2005; Potter et al., 2006) have been identified. Many of them cofractionates with lipid “raft” (Simons and Ikonen, 1997), a region enriched with cholesterol and sphingomyelin. However, it was unclear how apical signals interact with rafts because it is difficult to measure or quantitate specific interaction between lipids and proteins. For glycosylated molecules, proteins called galectins are known to bind them (Delacour et al., 2008; Brocca et al., 2022). Therefore, they are the only proteins involved in apical transport. However, not all apical proteins are able to bind galectins. Thus, the molecular mechanism for most apical proteins have long been elusive.

## The role of Rabs in apical transport

### Rab8

Rab8a is localized on the recycling endosomes and plays a role in budding of vesicles destined for the apical plasma membrane as described later (Nakajo et al., 2016). Rab8a has paralogues, Rab8b, Rab10, and Rab13. Rab8b, quite similar to Rab8a, is thought to be localized on the recycling endosomes. Rab10 is localized on the recycling endosomes as well (Khan et al., 2022). Rab13 is also known to be localized on the recycling endosomes in addition to the TGN and the plasma membrane (Ioannou et al., 2015). We discovered that apical transport was perturbed in the small intestine of knockout mice of Rab8a (Sato et al., 2007), which was previously reported to be necessary for basolateral transport in MDCK cells (Huber et al., 1993). However, this phenotype was limited to the small intestine and was not observed in other epithelial cells (kidney, trachea, retina, etc.). Rab8a expression was very high in the small intestine, suggesting its importance in the small intestine. This suggests that the Rabs involved in apical transport may differ depending on the type of epithelium. In addition, since abnormalities in apical transport in the small intestine are observed from about 2 weeks after birth, other Rabs (such as Rab11: see below) may be involved in apical transport until then. The 2-week postnatal period is the time when mice switch from breast feeding to solid food and when the digestive enzyme in the small intestine is replaced from lactase to sucrase. Therefore, it is likely that Rab8a is mainly involved in the apical transport of newly synthesized sucrase. Moreover, the intestinal phenotype of Rab8a knockout mice is very similar to that of a human disease, microvillus atrophy. When we sequenced the Rab8a gene from human patients, we were not able to detect mutations in this gene. However, when we stained the small intestinal tissue from a human patient, the amount of Rab8a was clearly reduced. Thus, we concluded some genes associated with Rab8a might be responsible for this disease (Sato et al., 2007). Though mutations in Rab8 have not yet been reported, the Myosin Vb gene, a binding molecule of Rab8, was mutated in a large proportion of patients of this disease (Müller et al., 2008). The phenotype was also confirmed in Myosin Vb KO mice (Cartón-García et al., 2015; Schneeberger et al., 2015). Myosin Vb has been known to bind to both Rab8 and 11 (Roland et al., 2007). In addition, Rab8, 11, and Myosin Vb KO mice show similar apical phenotypes in the intestine suggests that all of these are important for apical transport. The BBsome complex is important for cilia formation, and the presence of Rabin8, known as a Rab8 GEF, within the complex implicated a relationship between Rab8 and cilia formation. As cilia are localized on the apical surface, it is quite conceivable that Rab8 is involved in cilia formation as well (Nachury et al., 2007). A number of papers has been published stating that Rab8 is important for ciliogenesis (Yoshimura et al., 2007; Omori et al., 2008). However, since four molecules, namely, Rab8a, 8b, 10, and 13, belong to Rab8 family, we generated Rab8a+8b DKO mice to investigate whether there is functional redundancy among these Rabs. Contrary to our expectations, although the phenotype of DKO mice was more severe than that of Rab8a, no abnormalities were observed in cilia formation. Therefore, we further knocked down Rab10 and 13 in DKO MEFs and observed cilia. We found that cilia formation was perturbed in DKO + Rab10 KD (Sato et al., 2014). From the above, it became clear that Rab8a, b, and 10 are all required for cilia formation, and that if any of them remain, cilia can still grow. Regarding Rab10, though a previous observation in the intestine of nematodes has suggested that Rab10 is important for basolateral recycling (Chen et al., 2006), our results suggest that in mammals, Rab10 is involved in transport to the apical plasma membrane. In order to resolve this apparent discrepancy, we generated small intestine specific knockout mice of Rab10 because Rab10 KO mice are embryonic lethal (Lv et al., 2015). Preliminary data showed that there were no significant phenotypes in either the apical or basolateral regions. This indicates that the discrepancy results from difference in species.

It has recently been found that both Rab8 and Rab10 might be associated with the pathogenesis of Parkinson’s disease. Both of them are specifically phosphorylated by LRRK2, a responsible gene for Parkinson’s disease (Steger et al., 2016). There is a paper reporting that LRRK2 may at least affect recruitment of Rab8 and Rab10 to lysosomes to prevent lysosomal dysfunction (Eguchi et al., 2018). However, it is unclear to what extent this is related to cell polarity.

### Involvement of Rab8 binding protein in apical transport

**FIGURE 2:**
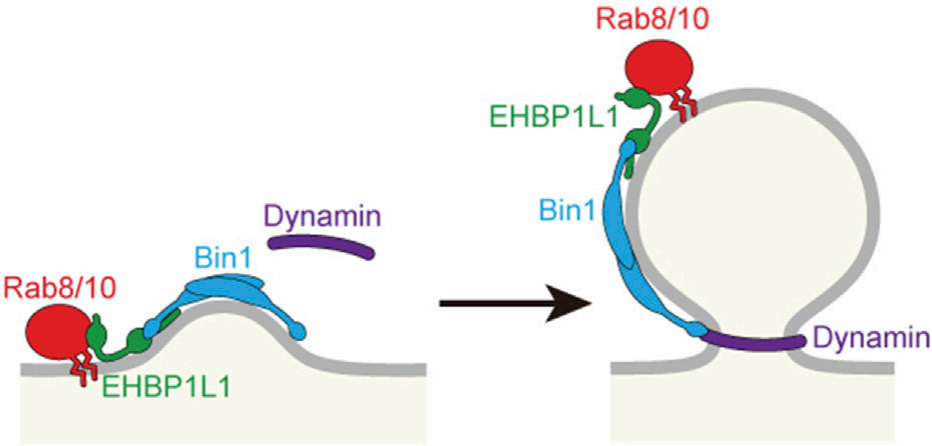
Budding at the recycling endosome to form a vesicle for apical plasma membrane. Proteins involved in budding are shown.

**FIGURE 3:**
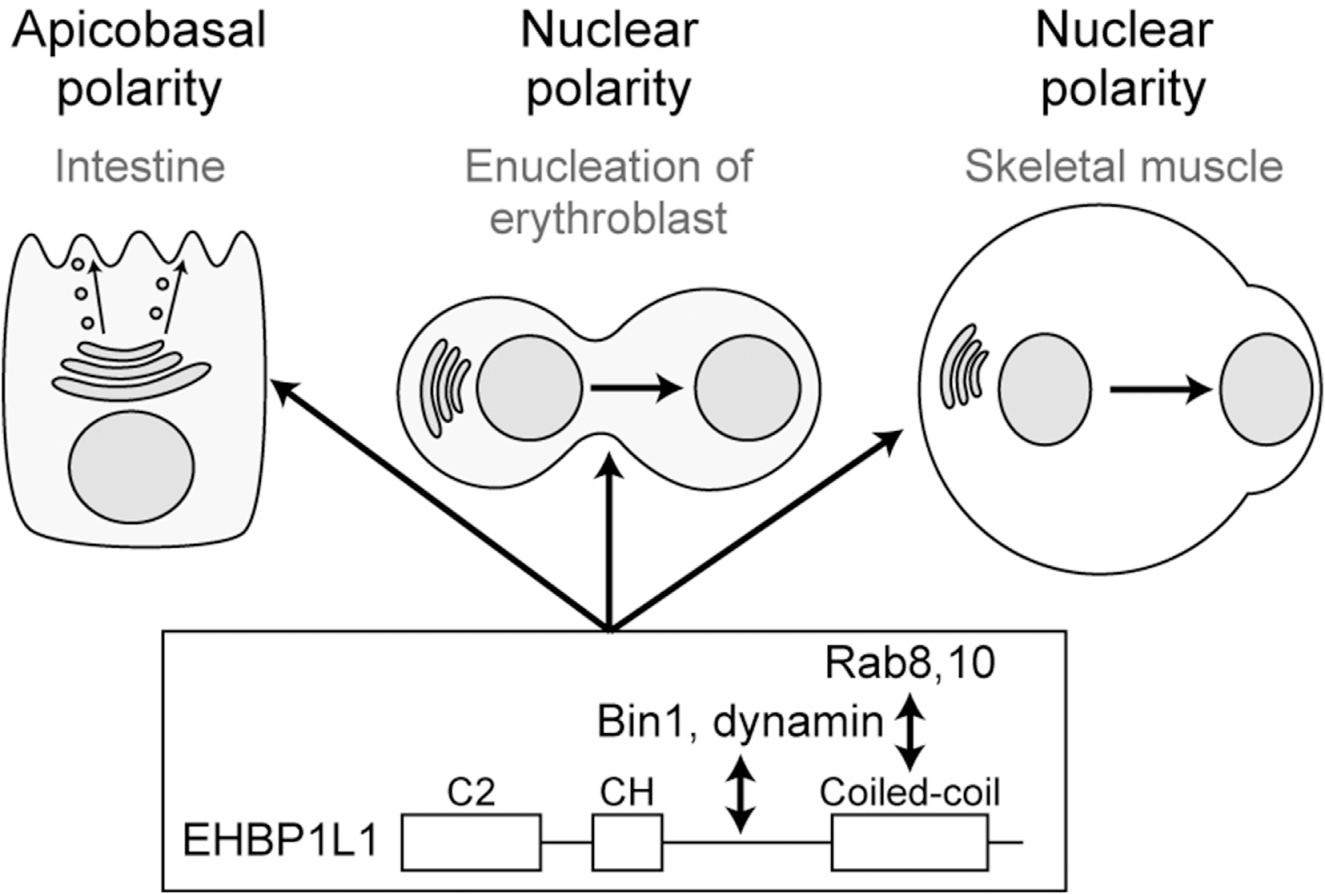
Rab8/10-EHBP1L1-Bin1-dynamin axis is involved in various polarization processes from apicobasal polarity to nuclear polarity.

To know the molecular mechanism of Rab8 in apical transport, we created a cDNA library of the small intestine and used the yeast two-hybrid system to search for Rab8a binding molecules. We discovered that a molecule called EHBP1L1 binds to Rab8a via its C-terminus (Nakajo et al., 2016). Furthermore, EHBP1L1 is localized to the recycling endosome with Rab8 and molecules involved in membrane deformation such as Bin1 and dynamin which bind to the proline-rich domain of EHBP1L1. It suggests that EHBP1L1 plays a role in the formation of vesicles from the recycling endosome to the apical plasma membrane. When EHBP1L1 KO mice were created, the length of the microvilli in the small intestine was reduced as expected. Interestingly, EHBP1L1 KO mice died within a few hours after birth due to severe anemia. When we examined the differentiation process of erythrocytes in the KO mice, we found that there was no abnormality in the differentiation capability to erythroblasts, although the final process from erythroblasts to erythrocytes was selectively impaired. This process is called enucleation in which the nucleus moves toward the plasma membrane and is expelled from the cytoplasm. In other words, EHBP1L1 was found to be important not only for epithelial apicobasal polarity, but also for the polarized movement of nuclei in one direction during the enucleation process (Wu et al., 2023). Nuclear polarization occurs not only during enucleation, but also during the differentiation process of striated muscle cells. While we were analyzing EHBP1L1, EHBP1L1 mutations were discovered in dogs with muscular dystrophy and anemia (Shelton et al., 2022; Jensen et al., 2022). The same phenotype in the striated muscles was also observed in our KO mice. The nuclei of the muscles did not localize at the periphery of the cell but remained in the center. Enucleation was inhibited not only by EHBP1L1 but also by KD of Bin1 and by dynamin inhibitors. However, the Rab involved was Rab10, not Rab8 probably because of the fact that Rab10 is much more abundantly expressed than Rab8 in erythroblasts. It is also worth noting that mutations in Bin1 and dynamin2 have been widely known as causative genes for human centronuclear myopathy (Jungbluth and Voermans, 2016; Hohendahl et al., 2016). Based on these facts, we propose that the Rab8/Rab10-EHBP1L1-Bin1-dynamin axis is necessary for cells to acquire polarity in a number of systems (Figure 3). In addition, considering that all of these gene products are localized to the recycling endosome (Nakajo et al., 2016) and that Rab11 is essential for apical transport as mentioned below, the recycling endosome is assumed to be important as a hub for apical transport (Figures 1, 2). To further investigate the function of EHBP1L1, we searched for binding molecules of EHBP1L1 and identified CD2AP (Iwano et al., 2023). CD2AP binds to molecules that regulate the actin cytoskeleton and the formation of cilia. This supports the idea that vesicles transported to the apical region interact with abundant actin filaments just below the apical plasma membrane. EHBP1L1 itself and its related molecule EHBP1 both have a domain called the calponin homology domain that has a potential to bind actin filaments. Therefore, it is possible that EHBP1, EHBP1L1, and CD2AP is involved in the formation of microvilli and the cortical plate where the actin filaments are abundantly localized by coupling the apical transport with actin polymerization and localization. In addition to EHBP1 and EHBP1L1, there are a number of proteins which have EH (Eps15-Homology) domain. Most of them also bind to Rab8 family. Among them, EHBP1 has been reported to be important for basolateral recycling in *Caenorhabditis elegans* together with Rab10 (Shi et al., 2010). However, since EHBP1L1 does not exist in nematodes, its role should be interpreted with caution. In addition, other EH domain proteins, the MICAL family, are known to bind to the Rab8 family, but there is currently no data showing their association with apical transport. At least, MICAL1 knockout mice showed no gross abnormalities (Van Battum et al., 2014). There were no reports for MICAL2, 3, L1, and L2 knockout mice.

### Rab11

Rab11 is localized to the recycling endosomes and is important for cargo recycling. It has been known that an increase in the area of the plasma membrane is necessary for bladder expansion, and that this is due to vesicle fusion in bladder epithelial cells (Khandelwal et al., 2008). Since it was known that Rab11 is necessary for this fusion process, we hypothesized that Rab11 may be necessary for the fusion of vesicles with the apical plasma membrane in epithelial cells in general. There are three genes in the Rab11 family: Rab11a, b, and Rab25, and it was known that the phenotype of Rab25 KO mice was mild (Nam et al., 2010), showing no abnormalities in apical transport. When we generated Rab11a KO mice, conventional (or systemic) KO was embryonic lethal. We generated small intestine-specific knockout mice and found that apical markers began to accumulate in the cells immediately after birth. This was earlier than the time when apical markers accumulate in Rab8a, b DKO mice (approximately 1 week after birth) (Sato et al., 2014) and Rab8a KO mice (approximately 2 weeks after birth) (Sato et al., 2007). This indicates that Rab11 is required for apical marker transport earlier than Rab8. To elucidate the molecular mechanism, we identified a novel effector of Rab11, RELCH. RELCH was found to be involved in non-vesicular lipid tranfer from the recycling endosome to the TGN rather than in vesicle transport (Sobajima et al., 2018). Thus, we did not observe its involvement in apical transport. Therefore, it is likely that Rab11 is involved in apical transport via a molecular mechanism mediated by other molecules, probably by Myosin Vb. It has been previously shown that the recycling endosome acts as a hub for transport from the Golgi to the plasma membrane (Ang et al., 2004), so it is likely that Rab11, like Rab8, is involved in some process in the recycling endosome for apical transport.

### The role of Rab8, 11 and its GEF in unconventional transport

Recently, Lin’s group has suggested that Rab8 and its GEF are important for unconventional transport in nematodes, and that apical transport is carried out by this pathway. Here, unconventional transport is defined as a transport that does not pass through the Golgi (Wang et al., 2022). Furthermore, the same group has reported a paper that Rab11 is also involved in this pathway (Li et al., 2024). However, it should be noted that the phenotypes of Rab8 KO in nematodes and mammals are slightly different. Thus, in mammals, the involvement of unconventional transport in the apical transport is still elusive.

### Rab6

Previous studies reported that Rab6 is mainly localized on the trans-Golgi and the TGN and is involved in both anterograde and retrograde transport from and to the Golgi apparatus in cells (White et al., 1999; Mallard et al., 2002; Grigoriev et al., 2007). In flies, it has been pointed out that Rab6 is important for transporting photoreceptors to the apical plasma membrane (Iwanami et al., 2016). In mammals, KO studies in MDCK cells and mouse embryos have shown that Rab6 is required for the anterograde and retrograde transport of basolateral proteins (Shafaq-Zadah et al., 2016; Homma et al., 2019). However, when we created a small intestine-specific knockout of Rab6a, no abnormalities were observed in the distribution of apical and basolateral markers in the small intestine (Iwaki A et al., 2020). Recently, we and the other group showed the importance of Rab6 for cell polarity in central nervous system (CNS). In CNS, two Rab6 paralogs, Rab6a and Rab6b, are expressed and CNS-specific deletion of both of them (Rab6 DKO) revealed that Rab6 is necessary for the apical transport of Crumbs3, a component of apical junctional complex, in neural progenitor cells (Brault et al., 2022). In addition, Rab6 DKO resulted in the loss of neuronal cell polarity. We found the accumulation of synaptic vesicle precursors (SVPs) adjacent to the Golgi apparatus in Rab6 DKO neurons. Therefore, we concluded that Rab6a+6b are involved in neuronal polarity through the regulation of axonal transport of SVPs (Zhang et al., 2024).

### SNAREs and their associated proteins in apical transport

We observed that syntaxin3, one of the t- (Q-) SNAREs, was essential for apical transport by analyzing intestine-specific conditional knockout mice (manuscript in preparation). Missense mutations in syntaxin3 have also been reported in some human patients with microvillous inclusion disease (MVID) (Wiegerinck et al., 2014). Since null mutant mice generated in our laboratory die soon after implantation (manuscript in preparation), we believe that human syntaxin 3 mutants retain some function. Munc18 is known to bind strongly to syntaxin and affect the binding of syntaxin to other SNAREs. In contrast to Munc18-1, a Munc18 family protein, which is abundant in the nervous system, Munc18-2 is localized on the apical surface of epithelial cells and is known to inhibit the binding of syntaxin3 to SNAP23 in *in vitro* experiments (Riento et al., 2000). A mutation in Munc18-2 (STXBP2) was also found in MVID patients (Stepensky et al., 2013; Dhekne et al., 2018), suggesting complex formation between syntaxin3 and Munc18-2 is essential for apical transport, particular, at the stage of fusion of apically transported vesicles to the apical plasma membrane.

Another candidate t-(Q-) SNARE involved in apical transport is SNAP23. This is supported by its apical localization in epithelial cells, and by the fact that amylase secretion from the exocrine pancreas is almost completely blocked in our acinar-cell specific KO mice (Kunii et al., 2016). As the exocrine secretion is performed by fusion of the secretory granules to the apical plasma membrane, SNAP23 is likely to be involved in this process. Interestingly, endocrine secretion of insulin is enhanced in pancreatic beta cell-specific KO mice. The main reason for this apparent discrepancy between endocrine and exocrine secretion is because SNAP25, orthologue to SNAP23, is expressed in addition to SNAP23 in the insulin-secreting endocrine cells. As SNAP25 is more competent in fusion than SNAP23, after SNAP23 depletion, remaining SNAP25 increases secretion efficiency (Kunii et al., 2016). We also showed that SNAP23 is necessary for apical transport of neural stem cells (Kunii et al., 2020).

So what are v-(R-)SNAREs? VAMP7 was previously considered to be required for apical transport in cells (Martinez-Arca et al., 2000). However, as VAMP7 KO mice show almost no abnormalities in cell polarity (Sato et al., 2011). It is known that VAMP8 is co-immunoprecipitated with syntaxin3 and SNAP23 *in vitro* (Pombo et al., 2003), and that VAMP8 KO mice, like SNAP23, show reduced pancreatic exocrine secretion (Wang et al., 2004) and reduced transport of AQP2 in the kidney collecting duct to the apical surface, resulting in hydronephrosis (Wang et al., 2010). In addition, our KD and KO studies revealed that VAMP8 is also required for apical transport of N-cadherin in neural stem cells (Kunii et al., 2020). Therefore, VAMP8 is likely to be most important for apical transport at least in exocrine glands and collecting ducts (Figure 1).

### Motor proteins

By screening using nematodes *C. elegans*, cytoplasmic dynein and dynactin components are important for the apical transport of recycling endosomes and the localization of apical markers (Winter et al., 2012). However, since the microtubules carrying cytoplasmic dynein run long distances, such as from the Golgi apparatus to the plasma membrane and from the apical membrane to the basolateral membrane, it is thought to be involved in long-distance transport within the cell. As mentioned above, Myosin Vb, crucial molecule for apical transport, is also a motor molecule. However, since it binds to actin, it is likely to be involved in short-distance transport from the subapical cytoplasm to the apical plasma membrane on actin filaments, which is abundant in the subapical cytoplasm.

### The role of glycans in apical transport

Galectin-3 is a cytoplasmic protein that binds to glycans, and there is an abnormal distribution of apical markers the intestine of galectin-3 knockout mouse (Delacour et al., 2008). Galectin-4 is also localized apically, but no major abnormalities have been observed throughout the body in KO (Brocca et al., 2022).

In addition, a number of apical proteins have been known to be heavily glycosylated. As previously observed, mutations in the glycosylation sites prevent apical transport (Potter et al., 2006; Vagin et al., 2009). Interestingly, heavy glycosylation generates membrane deformation to cause the membrane to generate protrusions (Shurer et al., 2019; Kuo and Paszek, 2021). Given these findings, glycans are likely to be involved both in the apical transport and in the formation of microvilli, apical protrusions at the plasma membrane. To further investigate the glycosylation process, we need the information how and where proteins and lipids are glycosylated in the Golgi apparatus. However, we still do not have sufficient information because of lack of antibodies and insufficient spatial resolution of light microscopy. As we have recently reported the endogenous localization and dynamics of various glycosylation enzymes (Harada et al., 2024), we will be able to get insights on this issue in the future.

### The role of lipids in apical transport

More than 20 years have passed since the raft hypothesis was proposed, which states that proteins transported to the apical region are selected by going to a compartment called raft, which is rich in sphingomyelin (SM) and cholesterol (Simons and Ikonen, 1997). Since then, the relationship between various lipids and polarized transport has been known. Recently, as a probe, Equinatoxin, that labels SM, has been developed, its distribution has become clear (Deng et al., 2016). In addition, a Golgi luminal protein, Cab45, is particularly abundant in SM-rich vesicles (von Blume et al., 2012). As it has been shown *in vitro* that Cab45 is important for transport to the apical region (Liu et al., 2023), analysis of the function of Cab45 *in vivo* is awaited. In addition to SM and cholesterol, the relationship with PIPs has been well studied. PI(4,5) P2 (Martin-Belmonte et al., 2007) and PI(3,4) P2 (Román-Fernández et al., 2018) have been shown to be enriched in the apical plasma membrane.

### Other candidate molecules involved in apical transport

FAPP2 (Vieira et al., 2005), annexin13b (Astanina et al., 2010), MAL (Takiar et al., 1995; Zacchetti et al., 1995), and MAL2 (de Marco et al., 2002) have been shown to be important for apical transport *in vitro*, but no phenotype regarding polarity was observed in knockout mice (Schaeren-Wiemerse et al., 2004; D’Angelo et al., 2013; Atik et al., 2014; Avriyanti et al., 2015; Venditti et al., 2019) (Table 1).

FAPP2 has been proposed to be involved in non-vesicular transport of lipids via membrane contact sites (MCS) (D'Angelo et al., 2007; D'Angelo et al., 2013). Recently, it has been found that OSBP, which is involved in non-vesicular lipid transport at the ER-TGN membrane contact site, is involved in polarized transport (Kovács et al., 2024), suggesting close relationship between membrane contact sites and polarized transport. Regarding the ER-TGN membrane contact site, it has been also reported that it is a source of calcium ions for activating Cab45, which is involved in the export of cargo from the TGN (Ramazanov et al., 2024), so in the future, we may have to pay more attention to the involvement of the MCS between the ER and TGN in polarized transport.

Recently, zebrafish has been frequently used for forward genetic screening. There are several advantages for using it. First, their intestines are easily observed from the outside, enabling large-scale forward genetics screening and live imaging. Second, their genes are more homologous to those of vertebrates than those of flies and nematodes. Studies of apical transport using zebrafish have already shown that acidification of the organelle lumen is important for apical sorting (Levic et al., 2020). An acidic luminal environment may promote clustering of O-glycosylated membrane proteins. Luminal O-glycans are a common sorting signal for apical membrane proteins. The authors speculated that V-ATPase–dependent acidification promotes neutralization of charges in O-glycans of apical membrane proteins and possibly also in glycolipids, thereby facilitating glycan clustering at the TGN. Alternatively, they considered pH may also influence binding of lectins such as galectin-3 which was shown to be involved in apical transport.

### The role of phase separation in apical transport

Phase separation or liquid-liquid phase separation (LLPS) has been drawn attention to explain many biological phenomena, including the biogenesis of secretary granule biogenesis (Parchure et al., 2022), unconventional protein secretion (Mendes et al., 2024), and short-distance synaptic vesicle transport at synapses (Qiu et al., 2024). Among them, it is interesting that phase separation by chromogranin B is presented to be involved in insulin sorting at the TGN (Parchure et al., 2022). As we mentioned in the previous section, discrete sorting signals have been elusive in a number of apical cargos. As LLPS does not need specific sorting signals, involvement of LLPS in sorting of apical cargos from cargos to other destinations should be considered in the future.

## Conclusion and perspectives

We have been studying cell polarity for more than 20 years, and the molecular mechanism of apical transport has made great progress since we first started. It is now becoming clear that apical transport by Rab8 and Rab11 families and their binding proteins play a major role in the molecular mechanism of cell polarity in general. In addition, they are likely to be involved in unconventional transport that does not pass through the Golgi. Based on their localization, we also revealed that the recycling endosome plays a central role in apical transport. However, there are still many to be investigated, in particular, about the sorting mechanism to the apical plasma membrane, such as the molecular entity of the apical signal. This will be a major challenge in the future. To do this, it is necessary to identify other molecules involved in apical transport. Our initial screening using nematodes was unsuccessful partly because many of the genes of nematodes and mammals were not so homologous. In the future, molecules involved in apical transport should be screened in mammalian experimental systems.

What was also unexpected was that the genes involved in apical transport are closely associated with many diseases. It was completely unexpected that Rab8 and its binding protein Myosin Vb are responsible genes for microvillous atrophy. In addition, phosphorylation of Rab8 and 10 may be associated with Parkinson’s disease. Rab8 binding proteins EHBP1L1, Bin1, and dynamin are the causative genes for anemia and centronuclear myopathy. These findings indicate the biological and pathological importance of the Rab8 family.

Another challenge is the relationship between apical transport and actin. The apical plasma membrane has microvilli which include a number of actin filaments. It has been questioned how microvilli are generated and what relationship is considered between microvilli and apical transport. As already mentioned, it is known that Rab8 effectors, Myosin Vb, EHBP1L1, and EHBP1, are likely to bind actin filaments. In addition, CD2AP, a binding protein of EHBP1L1, also binds to actin filaments. These findings suggest that apical transport is closely associated with actin filaments which are enriched in subapical region. However, how microvilli are formed still remains a mystery. In recent years, it has been reported that as glycosylation increases, fine protrusions like microvilli grow on the cell surface (Shurer et al., 2019). As already mentioned, it has been suggested that glycosylation is important for apical transport and that apical proteins are heavily glycosylated, so it is highly probable that glycosylation is involved not only in apical sorting but also in the morphogenesis of the apical plasma membrane. For this reason, it is important to revisit the apical sorting of glycans using a number of modern techniques. To do this, basic knowledge is required on the localization of glycosylation enzymes and raft in the Golgi apparatus and the recycling endosome and the molecular mechanisms underlying their localization. To achieve this, we need to utilize tools such as live imaging of fine structures using super-resolution microscopes and FIB-SEM, as well as techniques to identify the localization of endogenous molecules by knockin of genes using CRISPR/Cas9, and to identify binding molecules by proximity labeling using TurboID or APEX.
